# Optical Coherence Tomography in Retinal Detachment: Prognostic Biomarkers, Surgical Planning, and Postoperative Monitoring

**DOI:** 10.3390/diagnostics15070871

**Published:** 2025-03-28

**Authors:** Humza Zaidi, Jayanth Sridhar

**Affiliations:** 1 School of Medicine, University of Connecticut, Farmington, CT 06032, USA; 2 Olive View Medical Center, University of California, Los Angeles, CA 90095, USA

**Keywords:** retinal detachment, optical coherence tomography, photoreceptor integrity, swept-source OCT, OCT angiography, adaptive optics OCT, en face OCT, prognostic biomarkers, surgical planning

## Abstract

Retinal detachment (RD) is a vision-threatening ocular emergency that necessitates rapid diagnosis and intervention. This review examines the evolving role of optical coherence tomography (OCT) in RD by synthesizing the literature on preoperative biomarkers and advanced image modalities that inform diagnosis, prognosis, and surgical planning. We evaluated studies employing spectral-domain OCT, swept-source OCT, OCT angiography, adaptive optics OCT, and en face OCT to assess photoreceptor integrity, retinal detachment height, intraretinal cystic cavities, outer retinal corrugations and undulation, and macular involvement. The incorporation of OCT assessment into clinical practice may facilitate more precise surgical timing, technique selection, and postoperative monitoring. Further research is needed to standardize imaging protocols and validate specific prognostic biomarkers for optimal surgical outcomes. Key aspects include uniform imaging protocols, validating OCT-derived biomarkers such as ellipsoid zone integrity, and correlating OCT metrics with functional outcomes.

## 1. Introduction

Retinal detachment (RD) is characterized by the separation of the neurosensory retina from underlying retinal pigment epithelium (RPE), often leading to irreversible vision loss if not promptly addressed. As a vision-threatening pathology, RD may necessitate urgent retinal attachment surgery. RD affects between 6.2 and 17.9 individuals per 100,000 globally each year [1]. Rhegmatogenous RD (RRD) accounts for the majority of cases, resulting from liquefied vitreous entering through a retinal break, although tractional (TRD) and exudative (ERD) subtypes are present as well. While surgical techniques have improved, visual outcomes may vary widely and are difficult to predict [2,3,4,5,6,7,8].

Introduced in 1991, optical coherence tomography (OCT) has revolutionized our understanding of RD. Early time-domain systems have been superseded by spectral-domain (SD-OCT) and now swept-source OCT (SS-OCT) that achieve rapid scan speeds and axial resolutions as fine as 3 to 5 µm [1]. OCT is not only valuable for diagnosis but also for monitoring the stages of retinal detachment management, from pre-surgical evaluation through postoperative recovery. This review summarizes preoperative OCT biomarkers that predict visual outcomes; delineates the specific OCT features distinguishing rhegmatogenous, tractional, and exudative retinal detachments; describes intraoperative and postoperative OCT monitoring techniques; and discusses emerging imaging modalities and artificial intelligence applications.

## 2. Methods

We conducted a comprehensive literature search utilizing PubMed (U.S. National Library of Medicine, Bethesda, MD, USA) and Google Scholar (Google LLC, Mountain View, CA, USA), combining keywords such as “retinal detachment”, “OCT”, “prognostic biomarkers”, “OCT angiography”, and “adaptive optics”. Priority was given to clinical studies and reviews in English that addressed OCT findings in RD, including both rhegmatogenous and secondary detachments. We included evidence from randomized trials when available, as well as large prospective and retrospective studies that met relevance criteria. Many included studies are level II–III evidence (cohort or case series), since few randomized controlled trials exist on this topic. The strength and limitations of evidence are discussed where applicable. All key findings and statements are supported by references to the existing literature.

### 2.1. Prognostic OCT Features in Retinal Detachment

#### 2.1.1. Photoreceptor Layer Integrity

**Figure 1:**
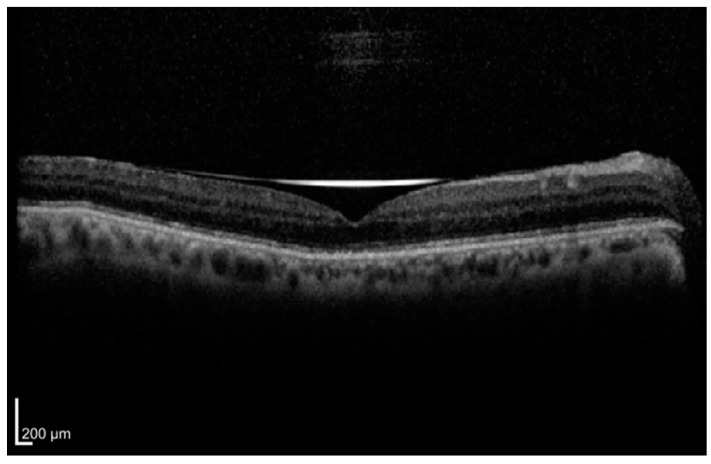
Ellipsoid zone disruption on OCT.

RD disrupts the normal physiological processes between photoreceptors and the RPE, leading to metabolic stress, photoreceptor swelling, and structural disorganization. Consequently, photoreceptor recovery after rhegmatogenous retinal detachment (RRD) repair relies on both the ability to withstand the initial metabolic insult and the subsequent reorganization following reattachment [9]. Given the critical role of photoreceptor integrity in visual outcomes, OCT has become a valuable modality for assessing retinal architecture over time. In particular, the integrity of the ellipsoid zone (EZ) and external limiting membrane (ELM) has been recognized as a robust predictor of postoperative visual function [10,11,12]. An example of EZ disruption on OCT is shown in Figure 1, where the EZ and ELM appear discontinuous beneath an attached retina after successful retinal detachment surgery. Noda et al. found that eyes (*n* = 42) with preoperative continuity of both the ELM and EZ had significantly better postoperative visual acuity than those with discontinuity of either or both layers [13]. Furthermore, in a retrospective analysis of 49 eyes, Guan et al. reported that persistent disruption of the EZ was strongly associated with worse postoperative visual acuity [14]. Sridhar et al. reported two patients with macula-off RD experiencing a postoperative improvement in VA secondary to EZ restoration [15]. These findings have been corroborated by other studies [16,17].

Recovery of EZ thickness and integrity have been associated with improved visual acuity following retinal reattachment. Longitudinal assessments utilizing en face OCT have demonstrated that the EZ exhibits a steady improvement in hyporeflectivity following RRD repair [18]. This decrease in hyporeflectivity—interpreted as an indicator of photoreceptor recovery—has been correlated with gains in ETDRS letter scores. Notably, en face OCT enables visualization of the entire EZ in a single instance, capturing many focal areas of hyporeflectivity more comprehensively than tradition cross-sectional B-scans [19]. Figueiredo et al. noted improvement in EZ reflectivity over a period of two to six years, with gradual recovery of the EZ correlating with incremental improvements in VA [18]. Abnormalities in the symmetry and circularity of the foveal avascular zone (FAZ) on OCT have also been described in RD [20].

The integrity of the cone interdigitation zone (CIZ) and the presence of a foveal bulge have been reported to be important predictors of postoperative best-corrected visual acuity (BCVA). For example, Ooto et al. demonstrated that mean cone density was significantly lower in eyes with a disrupted CIZ compared to those with an intact CIZ, while Park et al. found a borderline significant association between CIZ integrity and changes in postoperative vision [21,22]. Interestingly, it was also observed that the presence of a foveal bulge did not significantly correlate with BCVA changes.

#### 2.1.2. Height of Retinal Detachment

The height of retinal detachment (HRD) at the fovea refers to the distance between the inner border of the RPE and the outer border of back-reflection at the central fovea, or the vertical distance between the retinal base and detached fovea. It has been reported by multiple studies as a potential predictor of postoperative visual acuity (VA). Klas et al. found, in 102 eyes, that higher foveal detachment height was significantly associated with worse postoperative visual acuity at three months, with a critical cutoff of 500 μm, above which eyes had significantly poorer mean BCVA (*p* = 0.005) [23]. Zgolli et al. showed a greater level of macular detachment associated with lower pre- and postoperative VA [24]. Various other studies found a similar correlation between higher HRD and worse BCVA [25,26,27,28,29,30,31]. In a retrospective study of 47 eyes, univariate analysis demonstrated a correlation between HRD and VA—however, multiple regression analysis did not find this statistically significant [32]. Murtaza et al., in a meta-analysis of 17 studies (*n* = 910), found an overall weak correlation between HRD and postoperative VA [33].

#### 2.1.3. Intraretinal Cystic Cavities

**Figure 2:**
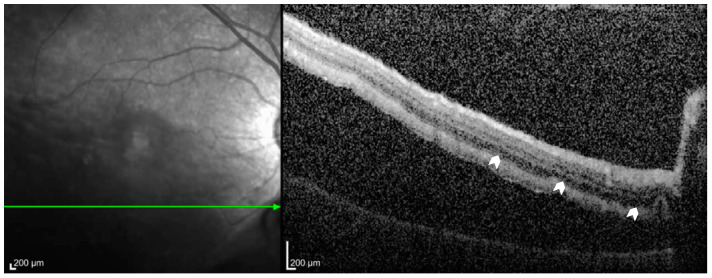
Intraretinal cystic cavitations on OCT.

Intraretinal cystic cavities (ICCs) are defined as well-delineated, hyporeflective spaces within the retinal layers that can be visualized using OCT, as seen in Figure 2 [34]. These cavities are thought to result from the accumulation of fluid secondary to a breakdown of the blood–retinal barrier, leading to localized edema [35]. They are frequently observed in cases of RRD. ICCs are often localized to specific layers, appearing predominantly in the inner nuclear layer (INL) or the outer nuclear layer (ONL), and in some cases, spanning both layers or extending into other retinal layers. When ICCs are confined solely to the INL and/or ONL, studies have generally found no significant correlation with postoperative VA [14,16,25,36]. However, investigations that included ICCs extending beyond these localized layers reported a significant association with worse postoperative VA [23,24,32,37,38,39,40]. Patients with preoperative ICCs that involved additional retinal layers exhibited a clinically significant decline in postoperative VA, averaging a loss of approximately two Snellen lines compared to those without such extensive cystic involvement [33]. These observations suggest that while limited intraretinal cystic changes may not substantially affect visual outcomes, more extensive cystic involvement indicates more severe retinal disruption, which in turn is associated with poorer functional recovery after surgical repair.

#### 2.1.4. Retinal Thickness

Several studies have examined the relationship between retinal thickness (RT)—particularly central macular thickness (CMT)—and postoperative VA [14,16,17,21,30,36]. CMT is defined as the distance between the internal limiting membrane (ILM) and the RPE at the fovea. Although one study has reported a significant correlation between CMT and postoperative VA in a cohort of 127 eyes [30], other investigations have not consistently found such an association [21,23,36]. This discrepancy may be attributed to variations in patient selection, surgical technique, and/or the timing of OCT measurements. Overall, the majority of evidence indicates that CMT alone may not be a reliable predictor of postoperative VA, but further research is needed.

In addition to CMT, other parameters of RT have been evaluated. Measurements of the ONL thickness, for instance, did not show a significant association with visual outcomes in multiple studies [21,41]. However, Gharbiya et al. found a statistically significant correlation between ONL thickness and BCVA [10]. In contrast, photoreceptor length—specifically, the length of the inner and outer photoreceptor segments at the fovea—has demonstrated a significant correlation with postoperative VA. Longer photoreceptor segments measured early after surgery have been associated with better final visual outcomes [37].

#### 2.1.5. Outer Retinal Corrugations

**Figure 3:**
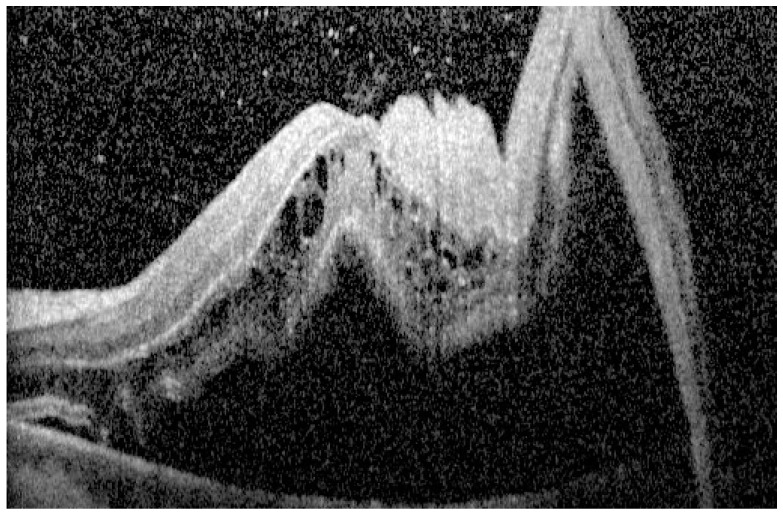
Outer retinal corrugations on OCT.

Outer retinal corrugations (ORCs) are characterized by high-frequency undulations, typically over three consecutive folds, occurring in the outer retinal layers, as seen in Figure 3. These corrugations are frequently observed in RRD eyes and appear to be related to the specific properties of the subretinal fluid. A multicenter study comparing 161 eyes (110 RD and 51 ERD) found that ORCs were present in 80% of RRD cases but were entirely absent in ERD (*p* < 0.001) [42]. This contrast may arise from a difference in the subretinal fluid osmolarity: the fluid in RRD is typically hypo-osmolar and comprises liquefied vitreous, which hydrates the outer retina, causing lateral expansion and subsequent folding. In contrast, ERD is characterized by inflammatory or neoplastic exudates that lack the osmotic properties that would induce such corrugations [42,43]. Nagpal et al. found the presence of ORCs to be associated with poor postoperative visual outcomes [44]. Abrupt changes in the reflectivity of the EZ have been associated with ORCs [45]. Case studies have shown that ORCs often resolve after successful retinal reattachment, correlating with improved retinal architecture and visual recovery. In RRD, the hypo-osmolarity of the subretinal fluid may overwhelm the RPE’s pumping capacity, thereby facilitating matrix hydration and fold formation [42].

#### 2.1.6. Outer Retinal Undulations

Outer retinal undulations (ORUs) represent a slightly different phenomenon and are defined by directional changes in the gradient of the outer retina. Specifically, ORUs are identified as having three or more directional changes across a 6 mm scan of the retina. In a retrospective study of 114 RRD eyes, ORUs were noted in 73.7% of cases with subacute detachments (symptom duration between 10 and 30 days), compared to only 38.5% in acute detachments (<10 days) and 27.3% in chronic detachments (>30 days) (*p* = 0.001) [17]. This pattern suggests that ORUs may serve as a marker of detachment duration, with a higher incidence in subacute cases.

Further supporting this concept, Yeo et al. observed that ORUs were more frequently detected in eyes with acute detachment and correlated with younger patient age as well as better postoperative visual acuity [17]. Their findings imply that these undulations may indicate a relatively short duration of detachment, when photoreceptor damage is potentially more reversible. However, a meta-analysis of preoperative OCT features using a random-effects model indicated that the overall quality of evidence regarding the prognostic value of ORUs is low, emphasizing the need for further robust research [33].

#### 2.1.7. Macular Detachment

The role of macular detachment in predicting postoperative VA has been examined. Multiple investigations have demonstrated that eyes with macular detachment tend to have worse postoperative VA compared to eyes with a spared macula [28,46,47,48]. This relationship is supported by the finding that the disruption of the outer retinal layers—especially in the foveal region—correlates strongly with diminished visual function. For instance, several studies have confirmed that the involvement of the macula is generally associated with poorer postoperative outcomes [17,23,36]. In contrast, one study did not demonstrate a statistically significant association between overall macular detachment and postoperative VA [47]. This finding raises the possibility that factors such as the duration of the detachment and/or the quality of surgical reattachment might modulate the impact of macular involvement on visual outcomes. It is plausible that in some cases, even if the macula is detached, timely and effective surgical intervention may allow for sufficient recovery of the photoreceptor layers to mitigate the functional loss. Poulsen et al. found that with macula-off detachment, intraretinal appearance also plays a role—with near-normal-appearing retina having a better visual prognosis than those with a disrupted appearance [28].

Mané et al. found that SD-OCT detected shallow macular detachment extending beyond the fovea in clinically diagnosed fovea-splitting RD [49]. Despite being anatomically macula-off, these cases had postoperative outcomes resembling macula-on, likely due to moderate preoperative visual loss and shorter duration of detachment allowing for restoration of the EZ and ELM [49].

### 2.2. OCT in Surgical Planning

#### 2.2.1. Spectral-Domain OCT and Swept-Source OCT

Both SD-OCT and SS-OCT have been widely utilized to evaluate macular changes in RD and after repair. SD-OCT is readily available and provides high-resolution imaging of the fovea, whereas SS-OCT offers deeper penetration and wider scans [50]. SD-OCT can be limited by its shorter wavelength source in cases of significant retinal elevation. SS-OCT is often more expensive and less widespread when compared to SD-OCT [50]. Bansal et al. identified five stages of retinal reattachment through SS-OCT monitoring after pneumatic retinopexy [50]. Initial stages involved rapid detachment height reduction, improved metabolic transport, and resolution of cystoid macular edema [50]. Subsequent stages included retinal contact with the RPE, photoreceptor deturgescence (normalization of outer segment volume), and restoration of outer retinal integrity, culminating in foveal bulge recovery. Faster recovery was observed in acute detachments with symptom duration under 24 h due to preserved outer retinal structure [50].

Ozsaygili et al. demonstrated that in eyes undergoing pars plana vitrectomy for macula-off detachments, the type of endotamponade used plays a crucial role [51]. They found that gas tamponade did not significantly alter retinal layer thickness on SS-OCT, whereas silicone oil was associated with pronounced thinning—particularly in the ganglion cell and outer nuclear layers—which correlated with poorer visual recovery. Notably, the degree of thinning in the ganglion cell layer emerged as a strong predictor of adverse outcomes [51]. Some researchers have reported that silicone oil tamponade leads to a reduction in the thickness of both the ganglion cell and inner plexiform layers, although the underlying mechanisms remain unclear [52]. Raczynska et al. further suggested that these specific retinal layer measurements could serve as prognostic indicators for final visual acuity [53]. Complementing these findings, Lee et al. observed that while silicone oil might cause temporary thinning of the parafoveal inner retina—with recovery noted after the oil’s removal—the thinning of the peripapillary nerve fiber layer appears to be more permanent, likely due to the mechanical pressure exerted by the tamponade [54]. Additional insights come from Horozoglu et al., who found that prolonged use of heavy silicone oil can achieve excellent anatomical reattachment, as evidenced by preserved ellipsoid zone continuity and maintained foveal thickness [55]. However, extended exposure to heavy silicone oil also increased the risk of developing epiretinal membranes [55].

Recent findings by McKay et al. underscore the importance of surgical technique on OCT-assessed retinal microstructure [56]. In a retrospective study of 300 macula-off RRD eyes, patients undergoing 23-gauge pars plana vitrectomy with subretinal fluid drainage through peripheral retinal break (PRB) or posterior retinotomy (PR) achieved significantly better VA compared to those treated with perfluorocarbon liquid drainage (PFCL). OCT evaluations showed that continuity of the ELM, EZ, and interdigitation zone was higher in the PRB and PR groups in contrast to the PFCL groups [56].

Despite these detailed imaging findings, there is still no consensus on the optimal surgical strategy for retinal detachment. While OCT provides critical information that may help tailor surgical approaches, current evidence is insufficient to definitively favor one method over another [57]. Some studies suggest that the surgical technique may influence postoperative integrity of the ellipsoid zone and external limiting membrane, as well as the development of outer retinal folds—which are associated with worse visual acuity and increased vertical metamorphopsia when these folds are closer to the fovea. Given the heterogeneity of surgical methods and tamponade agents, further comparative studies are necessary to determine the differential impacts on OCT biomarkers and visual outcomes.

#### 2.2.2. OCT Angiography

OCT-A enables noninvasive imaging of the retinal and choroidal vascular without the need for dye injection. However, OCT-A is susceptible to motion artifacts and segmentation errors, particularly in eyes with significant media opacities or residual subretinal fluid [58]. Recent advances in OCT-A have provided valuable insights into the microvascular changes that occur following RRD repair. One area of investigation has been the relationship between the outer retina and its underlying choriocapillaris. Hong et al. demonstrated that eyes with an intact outer retina maintained normal subfoveal choriocapillaris VD, whereas eyes with outer retinal defects exhibited a significant reduction in VD, which correlated with poorer visual acuity [58]. Changes in the FAZ and capillary plexuses have also been observed postoperatively. Chatziralli et al. reported that, following pars plana vitrectomy for macula-off detachments, there was an enlargement of the FAZ and a concurrent decrease in VD in both the superficial and deep capillary networks [59]. These vascular alterations were associated with thinning of the inner retinal layers in corresponding areas. Similarly, Xu et al. noted that while the superficial FAZ remained relatively unchanged after surgery, the deep FAZ exhibited significant enlargement, particularly in cases complicated by choroidal detachment [60].

It is believed that eyes undergoing surgery for retinal detachment exhibit an increase in FAZ area and a reduction in VD compared to normal eyes [57]. Although parafoveal VD shows some improvement over time, it often does not fully recover. Furthermore, eyes with a thinner preoperative foveal sensory layer tend to develop lower VD in the superficial capillary plexus after surgery. Nam et al. showed that eyes treated with pars plana vitrectomy exhibit a greater postoperative decline in VD than those managed with scleral buckling [61].

The effects of silicone oil tamponade on retinal microvasculature have been an additional focus of investigation. Roohipoor et al. found that eyes treated with silicone oil showed a significant reduction in VD within the parafoveal superficial capillary layer and across the total retina compared to normal eyes [62]. While parafoveal VD gradually improved over time, it did not return to preoperative levels. VD in the deep capillary plexus and choroidal flow also showed a decreasing trend in silicone oil-filled eyes—though the differences were not statistically significant. Interestingly, FAZ dimensions remained largely unchanged, and other studies have similarly reported that silicone oil does not have a major impact on macular VD or FAZ metrics.

OCT-A offers the advantage of quantifying retinal perfusion without dye injection, making it a powerful tool for postoperative monitoring of patients. However, its interpretation in the RD setting can be challenging—media opacities, distortion from surgery, and segmentation errors can all confound OCT-A images. Further research is needed to standardize its metrics (FAZ area, capillary density) in RD. Moreover, larger longitudinal studies would be beneficial to determine how OCT-A findings, such as reduced VD, translate into functional outcomes and whether interventions can improve microvascular recovery.

#### 2.2.3. Adaptive Optics OCT

Initial attempts to visualize photoreceptor changes following RD utilized adaptive optics integrated with fundus cameras. These studies revealed a decrease in cone density after RD, with partial recovery post-surgery [63]. However, the two-dimensional imaging provided by fundus cameras often resulted in overlapping signals from various retinal layers, creating artifacts that impeded accurate assessment of the cone mosaic. The advent of adaptive optics optical coherence tomography (AO-OCT) has addressed these limitations by enabling high-resolution, layer-specific imaging, thereby facilitating a more precise evaluation of photoreceptor integrity. However, AO-OCT is currently limited by its high cost, restricted availability, and longer image acquisition times when compared to other OCT methods [64].

A prospective study by Reumueller et al. exemplifies the clinical utility of AO-OCT in this context [63]. They examined patients undergoing vitrectomy with gas tamponade for macula-off retinal detachment, employing AO-OCT alongside standard spectral-domain OCT at 6 and 56 weeks post-surgery. While improvements in cone morphology were observed as early as 6 weeks after surgery, significant disruption of the cone mosaic persisted at the one-year mark [63]. These findings suggest that even when visual acuity appears satisfactory, subtle photoreceptor irregularities may underlie persistent visual disturbances. Beyond its diagnostic value, AO-OCT holds promise as a tool for refining surgical strategies. It can provide insights into the dynamics of photoreceptor regeneration and the restoration of the retinal microarchitecture, thus helping identify patients at risk for suboptimal visual recovery [64].

#### 2.2.4. En Face OCT

Unlike traditional cross-sectional B-scan OCT, en face OCT provides detailed, layer-specific views of the retina in the coronal plane. En face OCT may have utility in monitoring outer retinal folds, notable postoperatively after a vitrectomy [65,66]. It excels in visualizing subtle structural alterations, particularly in the outer retina. Additionally, it is more sensitive than B-scan OCT when detecting epiretinal membrane formation after RD vitrectomy, which has been shown to impact postoperative VA [67].

#### 2.2.5. Artificial Intelligence and Machine Learning

Recent developments in artificial intelligence (AI) are poised to greatly enhance the analysis of OCT imagines in RD. Machine learning—particularly deep learning utilizing convolutional neural networks—can be trained to detect subtle patterns in OCT scans that may elude human observation. A recent pilot study utilized a deep learning model on 6661 preoperative fundus images of RRD and was able to accurately predict the anatomic success of RD surgery [68]. Such a model, once refined, may assist the identification of high-risk cases that may need more aggressive studies. Similarly, Guo et al. developed a deep learning model utilizing OCT of the macula, age, gender, and preoperative VA that effectively predicted functional outcomes after rhegmatogenous RD repair in 184 patients (AUC of 0.91) [69]. Furthermore, AI can handle complex interactions of multiple imaging features and patient factors simultaneously—potentially uncovering composite biomarkers—wherein OCT patterns can be combined with clinical data to predict outcomes better than a single parameter. For example, Desideri et al. found, through an AI-driven analysis, that baseline visual acuity, ONL thickness, and the presence of hyper-reflective foci were among the strongest predictors of final vision in macula-off RD patients [70].

## 3. Postoperative OCT Monitoring

OCT serves as an essential adjunct in verifying RD surgery success and guiding early postoperative intervention when necessary. It can play a critical role in verifying anatomical reattachment after RD, particularly when media opacities such as corneal edema or vitreous hemorrhage obscure direct visualization [71,72]. Furthermore, while a recurrent detachment is usually evident on exam, OCT can confirm subtle subretinal fluid and pinpoint the RD location [73].

### 3.1. Persistent Subretinal Fluid

In the early postoperative period, OCT can detect residual subretinal fluid that persists in an otherwise attached retina [74]. Seo et al. report that 27–78% of eyes (*n* = 44) still have submacular fluid a month after successful buckling [75]. Although it is unclear if persistent subretinal fluid influences visual outcomes, several studies report delayed visual recovery [74,75] and others do not [76,77]. Serial OCT monitoring has shown that most residual subretinal fluid will gradually diminish over weeks to months [78]. Follow-up OCT showing new or expanding subretinal fluid raises concern for a recurrent detachment or missed retinal tear [79,80]. Thus, routine postoperative OCT surveillance is critical to differentiate innocuous residual fluid from pathologic and guide timely intervention.

### 3.2. Photoreceptor Integrity Recovery

In addition to tracking retinal attachment, postoperative OCT can be utilized to monitor the recovery of photoreceptor integrity. The reappearance and continuity of EZ and ELM lines is a favorable postoperative sign and has been shown to become clearer with three to six months following reattachment [18,81]. Hänsli et al. showed that eyes (*n* = 74) with an intact or recovering ELM on early postoperative OCT are more likely to achieve better visual acuity after six months [81]. Notably, photoreceptor recovery can continue for many years. Figueiredo et al. found a steady decrease in EZ hyporeflectivity over 24 months, with continued improvements up to four years, particularly in cases with shorter detachment duration [18]. Patients in this study experienced corresponding gains to ETDRS scores as the EZ normalized [18].

### 3.3. Cystoid Macular Edema and Epiretinal Membranes

Postoperative complications, including cystoid macular edema (CME) and epiretinal membrane (ERM), may also be identified early with OCT. Clinically, CME can be subtle, but OCT can show intraretinal cystic spaces [72,82]. A series of 130 eyes showed a CME incidence of 6.9% following buckle surgery [82]. ERMs have been shown to develop in approximately 10–20% of patients following repair, and OCT enables a sensitive approach for their detection [72]. If OCT follow-up showcases a progressive ERM that is impairing vision, surgical membrane peeling can be considered [83]. Pre-emptive internal limiting membrane peel in high-risk cases has been shown in a meta-analysis to reduce ERM formation by 88% [83]. Regardless of whether pre-emptive peeling is performed, postoperative OCT is critical for surveillance. OCT can also uncover proliferative vitreoretinopathy (PVR) changes early through shallow subretinal bands, folded retinal layers, or epiretinal sheets [71]. Recognizing PVR in its early stages through OCT enables earlier monitoring and reoperation if detachment recurs.

### 3.4. Demographic and Clinical Characteristics

The demographic and clinical characteristics of the patient may significantly influence OCT findings and overall prognosis, and these factors should be integrated into clinical decision-making. For example, older patients may have less capacity for photoreceptor regeneration, which could limit visual recovery despite successful reattachment. In contrast, younger patients often demonstrate more robust structural recovery on OCT and better functional outcomes [84]. A greater duration of detachment leads to further photoreceptor apoptosis and structural disorganization, whereas prompt repair allows for better visual outcomes [85]. Underlying ocular or systemic conditions also impact the appearance of RD on OCT. In diabetic patients with RD, OCT may reveal epiretinal fibrovascular membranes and a shallow, concave detachment profile with the absence of ORCs [57]. Highly myopic eyes may appear with a stretched choroid and possible choroidal detachment, as well as increased risk for persistent subretinal fluid postoperatively [57]. Future studies should systematically evaluate the influence of patient-specific factors on OCT biomarkers to enable more appropriate treatment strategies and postoperative management.

## 4. Conclusions

OCT has fundamentally transformed the clinical approach to retinal detachment by providing detailed insights into retinal microstructure and fluid dynamics that were previously unattainable. The integration of preoperative biomarkers—such as the integrity of the EZ, the presence of ORCS, and measurements of retinal detachment height—has markedly improved our ability to predict postoperative visual outcomes. Figure 1 summarizes these OCT-derived biomarkers for RD. Moreover, the advent of advanced OCT modalities, including SD-OCT, SS-OCT, OCT-A, AO-OCT, and en face OCT, has refined our understanding of the pathophysiological changes in the detached retina. These technologies not only enhance diagnostic precision but also inform surgical planning and postoperative monitoring, ultimately contributing to a more individualized treatment approach. Despite these advancements, challenges persist in the form of variability in the predictive value of certain imaging biomarkers and discrepancies arising from differences in surgical techniques and the use of various tamponade agents. This heterogeneity underscores the necessity for standardized imaging protocols and further validation of prognostic indicators in larger, well-controlled studies. Nonetheless, OCT remains an indispensable tool in the management of retinal detachment, offering critical guidance in both the timing and technique of surgical intervention as well as in the ongoing evaluation of retinal recovery.

## 5. Future Directions

Future research in the application of OCT to retinal detachment management should aim to address several critical gaps. Establishing standardized imaging protocols is paramount to ensuring consistency and reproducibility across clinical studies. This would facilitate more reliable comparisons of OCT-derived biomarkers such as EZ continuity, retinal detachment height, and the extent of outer retinal undulations. Large-scale, multicenter prospective studies are needed to validate the prognostic significance of these biomarkers and to develop robust risk stratification models that can guide individualized surgical strategies. Advances in high-resolution imaging techniques, including adaptive optics and en face OCT, promise to offer even greater detail in visualizing retinal microarchitecture; correlating these enhanced images with long-term functional outcomes will be an important step in understanding the dynamics of retinal healing and photoreceptor regeneration. Furthermore, OCT-A represents an exciting frontier in the evaluation of retinal and choroidal microvasculature post-detachment repair. Longitudinal studies focusing on vascular changes—particularly those involving the foveal avascular zone and capillary plexuses—may provide new insights into the relationship between vascular integrity and photoreceptor recovery. The integration of artificial intelligence and machine learning into OCT image analysis is also anticipated to enhance the accuracy and efficiency of detecting subtle retinal changes, thereby improving prognostication and informing tailored treatment approaches. Comparative investigations into the effects of different surgical techniques and tamponade agents on OCT biomarkers will be crucial in optimizing surgical outcomes and advancing our understanding of the mechanisms underlying successful retinal reattachment (Table 1).

## Figures and Tables

**Table 1:** Overview of multiple OCT-derived biomarkers in retinal detachment.

Biomarker	Description	Prognostic Implication
Photoreceptor layer integrity (EZ and ELM)	Continuity of the ellipsoid zone (EZ) and external limiting membrane (ELM) indicate preserved photoreceptor structure	Intact layers have been shown to correlate with improved postoperative visual acuity, while disruption predicts poorer outcomes [10,11,12,13,14,15,16,17]
Height of retinal detachment (HRD)	Vertical distance between the detached foveal retina and the RPE at the fovea	Greater HRD is associated with worse visual outcomes, although some studies report mixed findings [23,24,25,26,27,28,29,30,31,33]
Intraretinal cystic cavities (ICCs)	Well-delineated, hyporeflective spaces found within retinal layers secondary to fluid accumulation	Limited ICCs may have little impact, whereas extensive involvement predicts poorer visual acuity [14,16,23,32,37,38,39,40]
Retinal thickness parameters	Measurements of central macular thickness (CMT), outer nuclear layer (ONL) thickness, and photoreceptor segment length	While CMT and ONL findings are variable, longer photoreceptors segments have been linked to improved visual recovery [14,16,17,21,30,37]
Outer retinal corrugations (ORCs)	High-frequency undulations in outer retinal layers	The presence of ORCs has been associated with poorer visual prognosis, and may indicate a more severe retinal disruption [42,43,44,45]
Outer retinal undulations (ORUs)	Directional changes in the outer retinal contour	More common in subacute detachments, may serve as a marker for detachment duration and potential for reversible damage [17,33]
Macular detachment	Involvement of macula in retinal detachment	Macula-off detachment results in worse postoperative outcomes when compared to macula-on cases [17,28,46,47,48]

## Abbreviations

The following abbreviations are used in this manuscript:

AO-OCT	Adaptive optics optical coherence tomography
BCVA	Best-corrected visual acuity
CIZ	Cone interdigitation zone
CMT	Central macular thickness
ELM	External limiting membrane
ERD	Exudative retinal detachment
FAZ	Foveal avascular zone
HRD	Height of retinal detachment
ICCs	Intraretinal cystic cavities
INL	Inner nuclear layer
OCT	Optical coherence tomography
OCT-A	Optical coherence tomography angiography
ONL	Outer nuclear layer
ORCs	Outer retinal corrugations
ORUs	Outer retinal undulations
PFCL	Perfluorocarbon liquid
PR	Posterior retinotomy
PRB	Peripheral retinal break
RRD	Rhegmatogenous retinal detachment
RT	Retinal thickness
SD-OCT	Spectral-domain optical coherence tomography
SS-OCT	Swept-source optical coherence tomography
TRD	Tractional retinal detachment
VD	Vessel density
